# Supplementary material for: In Vitro Evaluation of the Probiotic Properties and Whole Genome Sequencing of Lacticaseibacillus rhamnosus J3205 Isolated from Home-Made Fermented Sauce
Source: Microorganisms. 2025 Jul 11;13(7):1643. doi: 10.3390/microorganisms13071643 (PMC12301047; doi:10.3390/microorganisms13071643)
Supplement: Supplementary file 1 [file microorganisms-13-01643-s001.zip › microorganisms-3623805-supplementary/supplementary/supplementary 2 0623 chen.pdf]

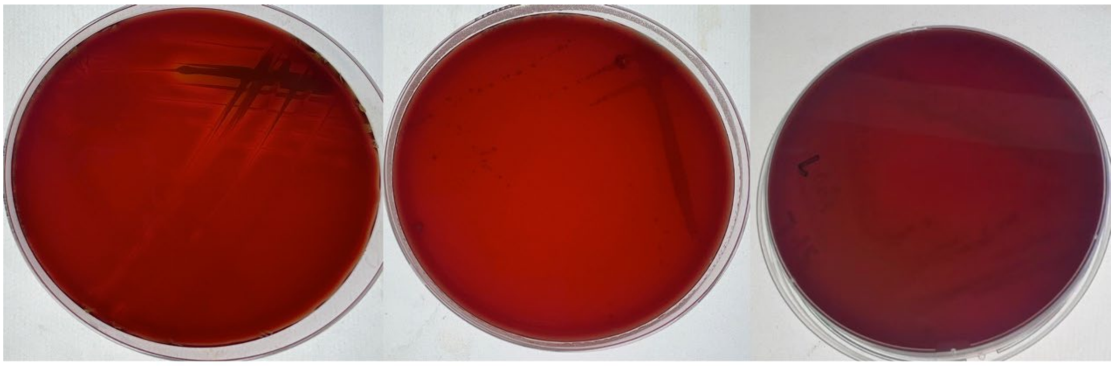

Figure S1. Hemolytic ability of *L. rhamnosus* J3205 and LGG: As a positive control, *S. aureus* ATCC 6538

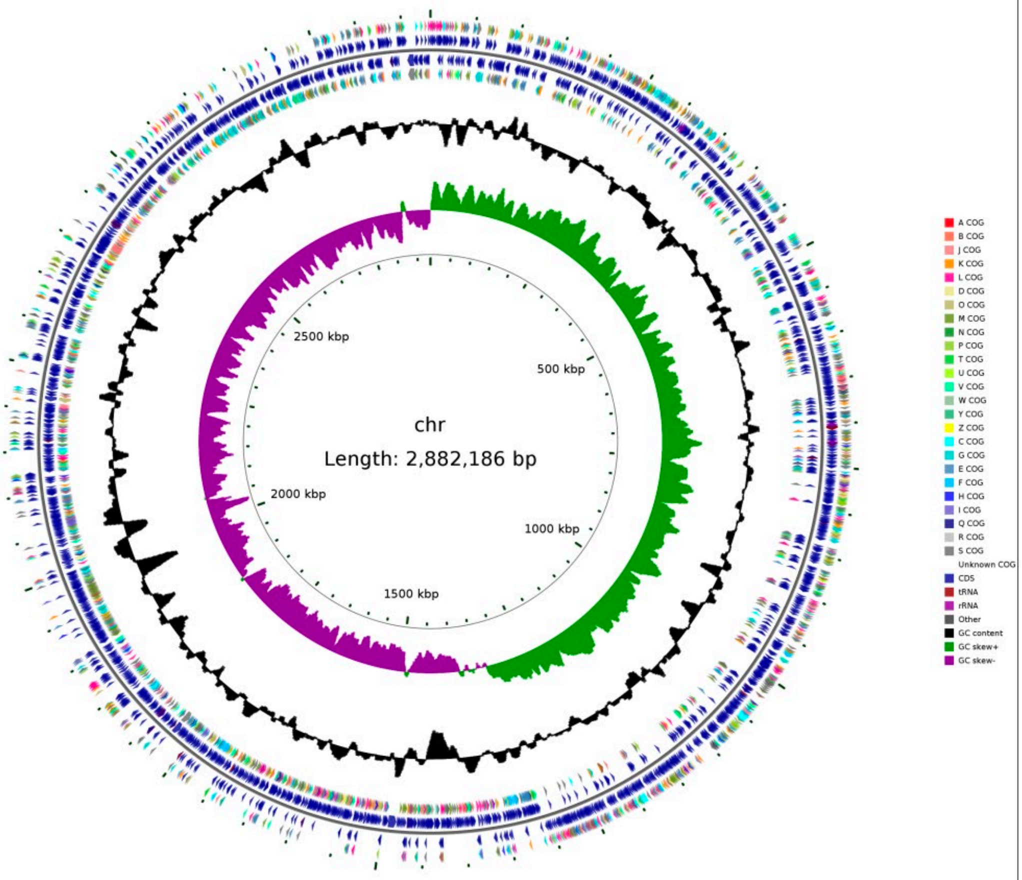

Figure S2. Genome circle map of *L.rhamnosus* J3205

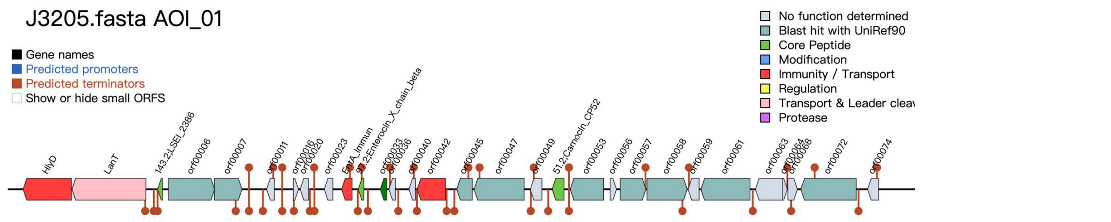

Figure S3. Bacteriocin information of *L. rhamnosus* J3205 based on the bacteriocin online software BAGEL4

Table S1. CRISPR/Cas within *L. rhamnosus* J3205 genome

CRISPR ID/Cas Type	Start	End	Repeat consensus
chr_1	775613	775714	GGTATAATGAAGTTGGAGATTGGAAGGGT TTTACCC
chr_2	2137462	2139015	GCTCTTGAACTGATTGATCTGACATCTACC TGAGAC
CAS-TypellA	2139036	2145198	csn2_TypeIIA, cas2_TypeI-II-III, cas1_TypeII, cas9_TypeII
chr_3	2386449	2386602	CGGCCATTGTGACTGAGGCCGCTTATGTTC CGGCTTACTCCCATAACGCGC

Table S2. Prophage regions of *L. rhamnosus* J3205

Region	Region Length	Completeness	Score	Total Proteins	Region position	GC%
1	31.9kb	Intact	150	26	1094398-1126394	45.08%
2	34.8kb	Intact	150	28	1565039-1599890	48.38%

Table S3. Insertion elements in the genome of *L.rhamnosus* J3205

Sequences producing	IS Family	E.value
ISLrh5	99.51	0
ISLrh4	100	0
ISLrh4	100	0
ISLrh4	100	0
ISLrh4	100	0
ISLrh4	100	0

ISLrh2	100	0
ISLrh2	100	0
ISLrh2	100	0
ISLrh2	99.94	0
ISLrh2	99.94	0
ISLrh2	99.94	0
ISLrh2	99.3	0
ISLrh2	99.23	0
ISLrh2	99.23	0
ISLrh2	99.23	0
ISLrh2	99.23	0
ISLrh2	99.23	0
ISLrh2	99.23	0
ISLrh2	99.23	0
ISLrh2	99.23	0
ISLrh2	99.23	0
ISLrh2	99.23	0
ISLrh2	99.23	0
ISLrh2	90.74	9.00E-08
ISLrh2	90.74	9.00E-08
ISLrh3	100	0
ISLrh3	100	0
ISLrh3	90.74	9.00E-08
ISLrh3	90.74	9.00E-08
ISLrh3	90.74	9.00E-08
ISLrh3	90.74	9.00E-08
ISLrh3	90.74	9.00E-08
ISLrh3	90.74	9.00E-08
ISLrh3	90.74	9.00E-08
ISLrh3	90.74	9.00E-08
ISLrh3	90.74	9.00E-08
ISLrh3	90.74	9.00E-08
ISLrh3	90.74	9.00E-08
ISLrh3	90.74	9.00E-08
ISLrh3	90.74	9.00E-08
ISLrh3	90.74	9.00E-08
ISLrh3	90.74	9.00E-08
ISLrh3	90.74	9.00E-08

ISLrh3	90.74	9.00E-08
ISLrh3	90.74	9.00E-08
